# Observing versus creating flowers: a review of relevance for art therapy

**DOI:** 10.3389/fnhum.2025.1504057

**Published:** 2025-01-30

**Authors:** Ephrat Huss, Mitsue Nagamine, Michele Zaccai

**Affiliations:** ^1^Spitzer Department of Social Work, Ben Gurion University of the Negev, Beersheba, Israel; ^2^Institute for Liberal Arts, Tokyo Institute of Technology, Tokyo, Japan; ^3^Department of Life Sciences, Ben Gurion University of the Negev, Beersheba, Israel

**Keywords:** arts-based research, observing versus experiencing, embodied aesthetic experiences, flowers, human’s relationship to flowers

## Abstract

This paper compares the embodied aesthetic experience of three types of images: photographed flowers, drawn flowers, and mandalas, summarizing data from three former comparative papers. The findings denote the strong embodied emotional connection of participants (changes in mood expressed in neural and physiological responses) to images of real flowers, as compared to the more cognitive reactions to drawings of flowers and cognitive stimulation of flower-like mandalas. These findings are discussed in terms of methodological relevance for art therapy and nature therapy. While it is known that flowers arouse positive emotions, this more nuanced comparison has interesting implications for visual art therapy, and for the therapeutic effects of nature photos, as opposed to drawn interpretations of nature.

## Introduction

Art therapy is concerned with creating embodied aesthetic experiences, within relational settings (Vaisvaser et al., 2024). Flowers are a “natural” example of experiences that can teach us about art therapy. The relationship between people and flowers is reciprocal because flowers “activate” humans to grow and to propagate them, creating a relationship with the flowers. In relational contexts such as giving and receiving them, flowers engage people in a multi-sensory experience that includes smell, texture, movement, and color. People form a caring relationship with flowers, actively planting, tending to them, and bending over to smell them and gently nurture them. Indeed, flowers are often dependent on this good care (Berger, 2008). Thus, flowers encourage people to interact skillfully in an embodied and caring way with the physical environment around them. All this embodied interaction makes them excellent examples and receptors for the experience of embodied aesthetics (Chemero, 2009; Shapiro and Spaulding, 2014; Varela et al., 2017). This is similar to how people “activate” art materials to create art in a relational setting in art therapy, where an intense relationship with art materials and with the therapist is activated.

While it can be argued that flowers may evoke this relationship because they can help food-growing possibilities and/or may be used as medicines (Hall and Knuth, 2019), since ancient times people have also grown ornamental flowers that have no evolutionary motivation. Humans have always had a strong relationship to flowers even when they provide no physical sustenance and when resources are low, pointing at the importance of their visual aesthetic experience for humans (Terry et al., 2020). This attraction to flowers is not purely on a survival level, since while flowers can provide some basic medicinal uses and serve as a sign of the fertility of the land, the major motivation for growing flowers seems to be aesthetic, just as we humans create art for aesthetic rather than survival reasons (Zhao et al., 2019). On all these levels, human interaction and relationship with flowers is an intense example of an embodied relational aesthetic experience that is often utilized in nature therapy, for example, where flower growing and tending is undertaken.

In continuation of the former ideas, we have looked at flowers as a positive relational aesthetic experience, that as such is therapeutic in of itself, in that flowers improve mood. People reported being happier receiving a large bouquet of flowers than when receiving other presents like a fruit basket or a candle (Haviland-Jones et al., 2005). Flowers were found to evoke feelings of compassion, less negative mood in the mornings, and increases in energy, happiness, and enthusiasm at work in individuals exposed to fresh cut flowers in their home (Haviland-Jones et al., 2005). This positive effect of flowers has also been used in art therapy to assist sexually abused children to express their feelings and thoughts, process their traumatic experience and reduce anxiety (Greenberg, 2001). The effect of flowers also has been apparent in mood related changes of physiological measures. For example, looking at fresh roses led to a decrease in Oxy-hemoglobin concentrations in the prefrontal cortex (Song et al., 2017) and an increase in parasympathetic nervous activity (Ikei et al., 2014; Ochiai et al., 2015), indicating physiological calming. Additionally, looking at peonies decreased systolic and diastolic blood pressure, heart rate, and pulse (Zhao et al., 2019), all indicators of physiological relaxation. Flowers were found to encourage people to make intimate connections among themselves (Guéguen, 2012; Guéguen et al., 2012) and behave in a more helpful way (Guéguen et al., 2015).

It is common knowledge that people react emotionally to external stimuli; however, flowers seem to elicit a special emotional response in humans, increasing positive affect, pro-social behavior, and affecting people’s judgment. With flowers, participants were better able to remember the room. A floral display was found to have a positive effect on feeling composed and confident (Adachi et al., 2000). However, interestingly, in our previously published papers, we found that this positive effect of flowers on people is also present when only observing a flower, rather than growing, cutting, and giving it to others: flowers have been shown to have a positive effect on peoples’ mood and their perception of others. With flowers present in the room, people judged pictures of other people more positively than without (Mojet et al., 2016). Additionally, flowers are also a relational, and not only aesthetic experience. This positive effect of real flowers, as well as photographs of flowers, has been shown to positively affect interactions between people (Haviland-Jones et al., 2005). In one study, positive emotions were maintained for three days after receiving flowers and made people likely to smile and create more social contact when given flowers (Fredrickson, 2000; Guéguen, 2011).

Interestingly, research has shown that concerning flowers, while popular colognes often have a floral top-note that will reduce negative emotions (Baron, 1997; Sarid and Zaccai, 2016), the visual components of flowers are the strongest, and people also grow flowers that do not have a scent, Pinker (1997). Indeed, fine art is full of images of flowers. Vision is a multimodal process that entails activation, not only of the visual areas of the brain, but also of sensory-motor, viscera-motor, and affective cerebral circuits (Ikei et al., 2014; Ochiai et al., 2015; Nadal and Skov, 2024).

What is it about flowers that pleasantly stimulate perception? Repeated, compositional elements of flowers such as color, shape, and pattern are repeated within the petal arrangement. They are also repeated within a group of similar flowers, providing the right amount of familiarity and innovation to calm but also to activate the brain. The familiarity engendered by symmetry in flower shapes may stimulate the brain just enough to remain alert but calm. This helps people to make sense of the world and improve their mood. This combined element of familiarity and surprise is a basic component of aesthetic experience that can move us emotionally (Ullman et al., 2002; Torralba and Oliva, 2003; O’Callaghan, 2008; Barrett, 2015). The Aesthetic experience thus enables a mindset of “flow” and deep concentration, and also mediates the ways that we communicate with others (Csikszentmihalyi, 1990; Malchiodi, 2012; Starr, 2023).

On the level of color, preference for color might have evolved because the various color channels are important in finding ripe fruit against a green background (Osorio and Vorobyev, 1996; Párraga et al., 2002). Plants with preferred colors and symmetry may become “superstimuli” for eliciting propagation and nurturing. On this level, perceptual elements of the flower connect to evolutionary theory through enhancing cognitive functioning. For example, due to overload of sensory information, humans look for coherent repeating patterns to organize their experience (Perls, 1980).

With respect to symmetry, it has been argued that humans have evolved with a preference for patterned symmetries because these can be detected easily as a recognizable signal within a wide variety of visual arrays (Enquist and Arak, 1994). In other words, we are attracted to symmetry. From this, the ease of recognition and the familiarity engendered by symmetry in flower shapes should be associated with improved mood (Zajonc, 1980).

The combination of clear shape and strong color, as well as smell, relative to the leafy background, enables the flower to be easily detected. This fits with the process of perception in which individuals group together elements into a coherent gestalt unit. For example, the brain perceives certain features that enable it to group together those parts that correspond to a single object, even if that object is partly hidden or seen against an unclear background (O’Callaghan, 2008). This involves reaching the simplest interpretation of incoming visual data using elements that are proximal, and that have a common visual property such as color or direction of movement. This principle of grouping elements together to fill in lack of information occurs in all the senses. This evolutionary need to separate figure from background (such as lion from jungle) can be practiced and fine-tuned, through seeing flowers that stand out as clearly different from their background (Barrett, 2015).

We can surmise that both the physical interaction with real flowers, and the visual experience of looking at pictures of flowers, activates multiple parts of the brain creating a stimulating perceptual experience. Indeed, this activation of multiple elements through visual perception is an aim of art therapy specifically (Hass-Cohen, 2003). Thus, we can speculate that while nature therapy may activate a relationship with a real flower, art therapy can utilize images of flowers to activate positive emotions.

These compositional elements of symmetry and variation, identified in flowers and in drawings of flowers, are also to be found in mandalas (Csikszentmihalyi, 1990; Hass-Cohen, 2003; De Petrillo and Winner, 2005; Malchiodi, 2012; Vaisvaser et al., 2024). Mandalas like flowers, are associated with improved mood due to a feeling of being able to make sense of the world. Through a repeating pattern that is understandable, yet also interesting and variable with changing shapes and colors that repeat. Mandalas are utilized intensely in art therapy and in arts and health, as a method to self-regulate mood, and to create a mindset of “flow” and deep concentration, as well as regulated communication with others (Ullman et al., 2002; Torralba and Oliva, 2003; O’Callaghan, 2008).

We have suggested the connection and similarities between real flowers, and art with flowers, and between mandalas, based on their perceptual elements. However, flowers and images of flowers (as compared to mandalas) also hold cultural and autobiographical memories that create a web of positive associations around flowers through former culturally contextualized experiences with them (Nelson and Fivush, 2004; O’Callaghan, 2008). While flowers universally elicit positive feelings, they have specific and culturally located sets of uses, rituals, and relational behaviors that connect to autobiographic memories and stimulate recall and accessibility of long-term memory (Ullman et al., 2002; Hampson et al., 2004; Nelson and Fivush, 2004; Bocanegra and Zeelenberg, 2009). Indeed, previous studies have shown that the mind explains what it sees through past experiences, for example in understanding depictions of perspective, or how a surface with low luminance is seen as painted dark or merely in a shadow (Ullman et al., 2002; Hampson et al., 2004; Nelson and Fivush, 2004; Bocanegra and Zeelenberg, 2009). This points to the multi-sensory element of flowers as enhancing episodic memory tasks. For example, a simple presentation of flowers, even a single flower, will release a strong and immediate positive reaction due to previous positive sensory memories of flowers. This is related to simple learned associations of flowers with positive social events, within the context of specific cultural construction of meanings. The activation spreading theory assumes that a sensory experience can be a trigger for additional experiences. Thus, real flowers and images of flowers can both stimulate the cultural memories that in turn serve as stimuli for new positive experiences (Nelson and Fivush, 2004; O’Callaghan, 2008).

We understand that autobiographical and cultural memory, and not only aesthetic experiences, are important for understanding the positive effect of flowers. These cultural contexts connect the person to their previous positive experiences including the learned associations of flowers. Flowers are often connected to positive social events, such as romance and celebrations, serving as mediating and transitional objects between people within the context of specific cultural constructions of meanings (Dryden, 1999).

In different parts of the world, cultural elements of flowers arouse different associations and there are cultural rituals created around them (Hurrell, 2016). For example, in Japan, cultural norms state that the person belongs to nature (macrolevel), so the person acts in ways that do not violate harmony with nature and feels bad when picking a flower is simply suggested (microlevel). The act of picking a flower takes on different meanings as a function of how relating to nature is culturally organized. In Israel flowers are associated with a strong familial and festive connection, such as bringing bouquets to mothers on Friday night, while in England, gifts of flowers have been associated more with romantic encounters (Eckerdal, 2017). A social-cultural theory claims that flowers are social signifiers. In different cultures, flowers are expected to convey sympathy, contrition, romance, celebration, or grieving (Heilmeyer, 2001). Flowers are also used to express religious feelings and in some religions are considered the direct route for spiritual communication (Stenta, 1930). The term “flower” is defined by Derrida as a “signified” word in Western culture, that is, as an especially dense holder of cultural meanings (Derrida, 2016). For example, they are a signifier of romance in Western culture (their petals, but also thorns, are part of this gestalt of romance) (Derrida, 1991). This is confirmed in the empirical research described above which shows that flowers enhance receptivity to romantic requests in Western culture (Guéguen, 2011). Similarly, Haviland Jones and colleagues have found that flowers enhance women’s positive emotions and social behavior (Haviland-Jones et al., 2005). Thus, for example in advertisements, images of flowers and real flowers are used as transitional objects to commemorate loved ones (Dimberg and Thell, 1988).

Both real flowers and pictures of flowers, elicit cultural memories, and enhance positive and relational associations. Mandalas, while compositionally similar, arouse different cultural memories. We have discussed in this work the tending of real flowers as an embodied aesthetic experience, and the observation of photographs of flowers as eliciting pleasant perceptual elements and cultural autobiographical memories. Thus, just looking at images of flowers can help to enhance mood (Huss et al., 2018; Huss et al., 2017). Maybe that is why they are so central in history of art and drawn so often, as one way to decorate homes.

We have shown how although flowers and images of flowers share compositional elements of symmetry-variation with mandalas, real flowers and images of flowers have specific positive cultural memories that enhance positive mood.

Our next conceptualization is to understand the difference between viewing two types of images of flowers: Artistic depictions of flowers versus photographs of real flowers.

The study of neuro-aesthetics focuses on understanding the physical and emotional response to creating and viewing art. Viewing art, as described by the embodied simulation theory is seen as a relational encounter between the viewer and the remnants of the creator, thus it is an intersubjective experience, through which two conscious beings meet (Ji, 2020; Urakami et al., 2022). Artists have always used nature as a base or as a source of aesthetically pleasing elements from which to create works that reflected their inner feelings, emotions, and beliefs, and to communicate them to the viewer (Joshi et al., 2011). Thus, photographs of flowers and artwork depicting flowers, create different aesthetic experiences. This paper, explored similarities and differences between real flowers, images of flowers, mandalas, and artwork depicting flowers. What can we learn from this comparison for art therapy? How can we explain the neurological and phenomenological difference between the physical interaction with actual flowers, as in nature therapy for example, and the use of photographed flower images, and the observation of artistic depictions of flowers within art therapy? Can this teach us something about the act of creation versus the act of observation? In other words, is it enough to look at images, or does one have to be involved in creating them? What is the difference between creating art and observing art?

To consider this specific question more deeply, we will now review three previously published papers of ours about man’s relationship to photos of flowers, photos of mandalas, and photos of fine artwork.

In our research, we tried to understand how people experienced black and white photographs of real flowers, in terms of perceptual elements and cultural memories. We also asked, can these images of flowers also elicit the multiple responses we saw in reaction to photographs of real flowers? Can these images enhance relational connections to the world, enhance flow, and aesthetic pleasure? Can they stimulate positive autobiographical cultural and relational memories? And can they elicit a stance of caring for and interacting positively with flowers and the world?

While the full methodology of these research studies can be read in the papers themselves, here we will describe the main points of the experimental setup and the analyses of the data. We used a combination of quantitative and qualitative approaches. The participants were either university students, equally distributed across gender (Huss et al., 2017, 2018) or adult participants from 18 different countries, with a majority of women (189) over men (46) (Urakami et al., 2022). For the quantitative analyses, the participants were asked to rate black and white photos of flowers and of non-filled in mandalas (Huss et al., 2017, 2018), flowers, nature scenes, and flower drawings (Urakami et al., 2022). Statistical analyses included Generalized Mixed Models, *T*-tests, chi-square and Anova. For qualitative assessment, we used a concept group (Huss et al., 2017) and collected free associations of the participants about the pictures the viewed (Huss et al., 2017, 2018, Urakami et al., 2022).

Following are our findings:

### Real flowers and images of flowers as creating an overarching positive reaction

We found that while flowers have strong cultural signifiers, (even without going into issues of gender and class) there is an overarching pleasure in flowers that is based on a combination of calmness and arousal, that seems to emerge from their perceptual characteristics, even when seen in a photograph as compared to caring for and smelling an actual flower, for instance. This points to a more universal conception of “floweriness” expressed by the overarching positive reactions of participants toward all flowers (Huss et al., 2017). These positive associations were also apparent when people were shown black and white images of flowers. We understood this, based on our findings, as happening due to an overarching connection to flowers that transcends cultures, a deep environmental element (Kopytin, 2022).

### Flowers as creating better mood, and mandalas as creating more interest

Based on our findings, we hypothesized that if the perceptual element of flowers is so strong, we would expect images of symmetrical flowers and mandalas, to create the same reactions. We continued to compare participants’ reactions to a mandala, another symmetrical and round object that is drawn, and that has the same visual characteristics of repetition, variation, color, and round shape that were found to create the experience of “flow” as described here. In other words, if it is the visual compositional elements of flowers that elicit positive reactions, we expected the mandala would do the same. For this comparison we chose a black and white symmetrical flower similar to a mandala. We asked the participants to give a grade from 0 to 5 concerning several parameters comparing their reaction to the mandala vs. the photograph of the symmetrical flower image that was presented next to it.

We compared the emotions induced by a photo of a radially shaped flower and by a photo of a round mandala, two items with similar symmetry and shape. The flower induced significantly (*p* < 0.01) more happiness than the mandala and the mandala was perceived as significantly more interesting than the flower (Huss et al., 2017).

The findings point to similarity in terms of prettiness and interest between the photos of the flower and the mandala. However, the flower was ranked significantly higher than the mandala for happiness levels and for calmness, respectively. When we compared a round flower with a round mandala, statistically significant differences in the measurement of happiness, calmness, and interest were found between the two, implying that shape was not the central attracting factor of the flower. The flower aroused more happiness than the mandala, although they were compositionally similar.

Interestingly the mandala aroused more interest than the flower, maybe because of its visual complexity. In other words, the compositional complexity of the mandala aroused more cognitive interest, but this did not necessarily create more feelings of calm and happiness, feelings which were induced by the flowers.

### Artwork of flowers as creating less positive mood and creating a more cognitive reaction to the quality of the art

After exploring the relationship between images of flowers and mandalas, we moved on to explore the effect on people of artwork with flowers as the subject. While both of these elements are drawn, they are different in that one is an expressive representation of a flower and the other is a flower-like compositional constellation, that is not a flower. How do these artworks of flowers influence emotions, cognitions? And overall experience? In this comparison, we wanted to understand if emotional response of people is different when viewing photos of flowers compared to viewing drawing of flowers? In other words, we wanted to check if the idea or abstraction or aesthetic quality of a flower is what elicits a reaction, or an image of the flower itself (as in a photograph). Our previous studies have revealed basic differences in the perception and induction of emotions engendered by photos of flowers as compared with drawing of flowers. In the study, photos of flowers elicited significantly (*p* < 0.001) more excitement, awareness, and pleasantness than drawings of flowers, which were also denoted as “boring” by 38% of the participants. Overall, the photos induced stronger positive feelings than the drawings (Urakami et al., 2022).

We observed that art images of flowers reduced the positive emotions and created a switch to a more cognitive rather than emotional level, focusing on the merit of the art rather than the flower. The artwork did not, in other words, arouse an embodied aesthetic experience or a set of cultural associations, but rather aroused an intellectual evaluation of the level of the art product itself. These reactions were the same to all types of art pictures regardless of variation in style of the art images used. Also, interestingly, many comments were about the art medium, rather than the content, the flower, such as that the paint was thin or thick, such that the flower element was subsumed inside the art element. People described the images as interesting or boring, as clever or kitsch, without relating to the flower element depicted. In other words, it seems that the medium was a more dominant factor than the subject of flowers. Viewing flower photos was rated as being more pleasant than viewing flower drawings.

Flower photographs were rated as making the participant feel more excited and awake as compared flower drawings. In general region, age, and gender did not seem to affect liking and emotional ratings. The participants’ emotional reaction toward photos of flowers were stronger compared to flower drawings and made them feel more excited and awake. Results suggest that the participants’ emotions and feelings toward photos of flowers were stronger compared to flower drawings and made them feel more excited and awake as well as evoking stronger positive feelings.

## Discussion

We will start with a summary of our former central findings of our comparisons as elaborated in this paper, and we will then discuss them in light of their relevance to art therapy: Real flowers and images of flowers arouse an overarching positive reaction, flowers creating better mood, and mandalas creating more interest, artwork of flowers creating less positive mood and creating a more cognitive reaction to the quality of the art. How can we explain these differences, and what can we learn about them for use in art therapy?

We can theorize that something in the idea or gestalt of a flower, from an ecological perspective, even in a small photograph, manages to arouse a basic recognition or excitement that goes beyond aesthetic appreciation and appeals to the senses. One can imagine from this that maybe people are indeed deeply embedded in, and thus aroused by, a deep basic connection to nature (Berger, 2008) together with, the autobiographical and cultural memories of positive relational interaction described above (Chemero, 2009; Shapiro and Spaulding, 2014). These cultural and basic connections are lost when looking at mandalas which arouse interest and engagement, but less positive emotions. In other words, the connection to flowers goes beyond compositional elements. It also explains the gap between artwork of flowers, that lose the sensory immediacy of flowers as a symbol of many cultural meanings.

What do these findings teach us about art therapy? This finding is interesting in light of the common use of mandalas to create calm and positive emotions in art therapy interventions. From this review of the research, we can learn that perhaps we should be using mandalas to arouse people and activate cognitive engagement, while using flowers and images of flowers to promote feelings of calm and positive feelings. In other words, mandalas could be good to use in situations of neurological difficulty, traumatic freeze, shut down, or depression, while interactions with images of flowers, more directly improve mood, in situations of health difficulty or low mood.

Additionally, it is important to remember that in art therapy people observe images, but mostly, “create” images. This act of creating images, once again involves a strong relational element, due to the art therapist moderating the interaction with the art. Creating an image also includes a strong embodied element, due to the manipulation of physical materials, and the creation of something new with them. Thus, art therapy is closer to the “caring” for a “real” flower, than to the viewing of an “image” of a flower. This also has implications for understanding the therapeutic quality of crafts and of art processes. In crafts one engages deeply with materials to create an aesthetic product, often with interchangeable elements of repetition and variation. Most importantly, the aim is often to create something “for” someone, in a relational context of care. However, we saw that even looking at photographs of flowers, enables a strong sense of connection and improvement of mood. Further research could explore the “creating” of flowers in art, as compared to the “observing” of flowers.

Concerning nature therapy, we gain new insights into the intense power of observing and tending flowers, and the power of images of flowers when one cannot be in nature (for example, when confined due to illness or in a prison). A limitation could be the explorative preliminary nature of this research, that wound along a path from flowers to mandalas to artwork. However, this has created an interesting conceptual map that can be further explored in future work on each of these levels, as each stage also validated the former stages.

This study found that a small black and white photo of a flower can elicit a set of embodied relational and cultural associations, and shift mood. Future research can more deeply explore these perceptual differences as they relate to the different stages of arts therapy, including interacting with, creating, and also observing images.
